# A novel approach to glaucoma screening and education in Nepal

**DOI:** 10.1186/1471-2415-8-21

**Published:** 2008-10-26

**Authors:** Suman S Thapa, Kurt H Kelley, Ger V Rens, Indira Paudyal, Lan Chang

**Affiliations:** 1 Nepal Glaucoma Eye Clinic, Tilganga Eye Centre, Kathmandu, Nepal; 2 University of Vermont/Fletcher Allen Health Care, Burlington, VT, USA; 3 Department of Ophthalmology, Vrije Universiteit Amsterdam, Amsterdam, The Netherlands; 4 University of Michigan, Ann Arbor, MI, USA

## Abstract

**Background:**

Glaucoma is a major cause of blindness worldwide and an increasingly significant global health problem. Glaucoma prevention and management efforts have been challenging due to inherent difficulty in developing a simple and cost-effective screening plan, limited access to health care and educational resources, poverty, and inadequate knowledge of the disease, particularly in developing countries. Starting in 2004 the Tilganga Eye Centre in Kathmandu, Nepal has provided targeted glaucoma screening, treatment, and education through a combination of clinical outreach programs and educational activities for patients.

**Methods:**

A simple, age-based glaucoma screening algorithm was incorporated into three one-day cataract screening clinics. Using this algorithm, patients who were newly diagnosed with glaucoma were referred to TEC, where medication and surgery were provided free of charge through private donor funding. In addition, we describe two ongoing educational programs for increasing glaucoma awareness: an annual Glaucoma Awareness Week (which includes free screening, treatment, and counseling), and a repeating lecture series which generates new counselors.

**Results:**

From 2004 to 2007 screening at the annual Glaucoma Awareness Week resulted in the diagnosis of 120 individuals with glaucoma, or 7.6% of total registrants. Attendance increased annually with a trend toward an increasing number of returning patients but a decreasing percentage of newly diagnosed patients, though the absolute numbers have remained relatively stable (range 21 to 38). Data from the three one-day screening clinics in 2006 show that approximately 2 to 4% of patients 50 years of age or older per clinic were newly diagnosed with POAG.

**Conclusion:**

This multi-faceted approach appears to successfully identify individuals with glaucoma and provide treatment to those who would otherwise not be able to afford it. While more data is needed to validate this model, specifically regarding the effectiveness of educational activities, long-term visual outcomes, and medication compliance, it may serve as a useful framework for other developing countries with similarly limited resources.

## Background

The term glaucoma encompasses a group of ophthalmic diseases that are believed to share the common pathophysiology of elevated intraocular pressure (IOP), or abnormal sensitivity to high-normal IOP, resulting in damage to the nerve fiber layer of the retina and irreversible vision loss [1]. The two most common forms of the disease are primary open angle glaucoma (POAG) and primary angle closure glaucoma (PACG), with variable patterns of disease prevalence in different ethnic groups[2]. The World Health Organization (WHO) reported in 2002 that glaucoma accounted for approximately 4.6 million cases, or 12.3%, of the 37 million cases of blindness worldwide, making it the second most common cause of blindness after cataract[3]. Furthermore, it has been estimated that over 60 million people worldwide will have glaucoma by 2010, resulting in 8.4 million cases of bilateral blindness[2]. These figures plainly suggest that glaucoma is a disease of particular public health significance; indeed, blindness and low vision, independent of cause, are important global health issues because they confer increased morbidity and mortality, decreased quality of life, and substantial economic productivity loss [4-8]. Due to the irreversible nature of the vision loss that occurs with glaucoma, management strategies must focus by necessity upon early detection and prevention of disease progression through strict control of IOP. However, these goals are limited by the fact that a simple and cost-effective screening plan for glaucoma has yet to be developed [9-12]. The prevention of glaucoma in developing and developed countries alike is further hindered by factors such as limited access to health care, poverty, limited formal education, and inadequate knowledge or understanding of the disease [13,14].

The country of Nepal faces these obstacles on a significant scale. The United Nations 2006 Human Development Report ranked Nepal 138th out of 177 countries based upon its human development index (HDI) which takes into consideration life expectancy as well as indicators of education and standard of living [15]. In addition, Nepal's predominantly mountainous terrain (which comprises the northern two-thirds of the country) and variable weather conditions contribute to a total road network that is the lowest in the region according to the World Bank, with only 36% of the population having access to all-weather roads and the health care facilities that they may lead to[16]. According to estimates from the Nepal Blindness Survey (conducted from 1980 to 1981), glaucoma accounted for 3,820 cases, or 3.2%, of bilateral blindness in Nepal[17]. More recent WHO regional data (including Nepal, India, Pakistan, and Bangladesh) places the number at 9%2; notably, neither figure accounts for the presumably greater number of individuals with less advanced disease. To address this growing problem, the Tilganga Eye Centre (TEC), a tertiary eye hospital located in Kathmandu, Nepal, initiated a multi-faceted approach to glaucoma screening, treatment, and education, specifically through the incorporation of glaucoma screening into established clinical outreach programs and educational activities with referral for treatment as needed. This model may be of interest as a framework for other developing countries where health care and educational resources are similarly limited.

## Methods

### 1. Screening clinics

**Figure 1:**
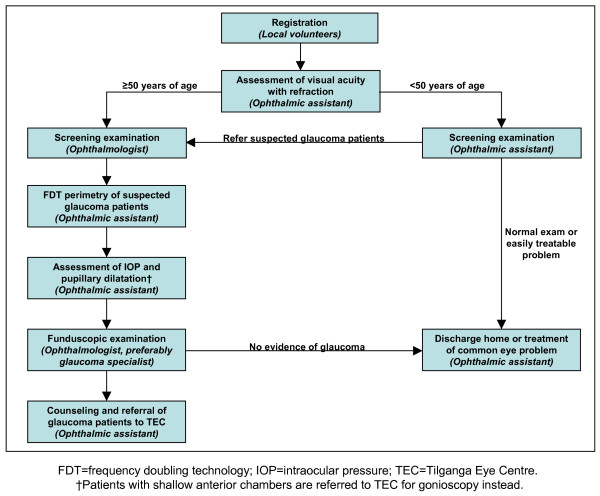
**Patient encounter algorithm utilized at one day screening clinics**. FDT = frequency doubling technology; IOP = intraocular pressure; TEC = Tilganga Eye Centre. †Patients with shallow anterior chambers are referred to TEC for gonioscopy instead.

TEC conducts two different types of outreach programs: microsurgical eye clinics (almost exclusively cataract) in remote areas of Nepal and neighboring countries that typically last several days, and one-day cataract screening clinics in villages closer to the capital of Kathmandu that are accessible by road. Made possible through the financial support of private donors, these programs target patients who have not sought out care due to limited or non-existent local health care facilities, financial constraints, or simply lack of awareness. In anticipation of the one-day screening clinics, local organizers are recruited and invited to TEC the week prior to the clinic for instruction and education about the objectives of the clinic. These organizers are responsible for advertising the clinic (e.g. through posters, flyers, or door-to-door visits), managing volunteers, procuring an appropriate facility, and the transportation of patients to TEC. In turn, TEC provides a team of ophthalmologists, ophthalmic assistants who have completed three years of university-affiliated training, free medication, and diagnostic equipment such as portable slit lamps. The clinics represent excellent opportunities for the incorporation of "opportunistic" glaucoma screening, which was performed at three different one-day clinics and facilitated by the addition of a Perkins applanation tonometer (Haag-Streit^®^) and frequency doubling technology (FDT) perimeter (Humphrey/Zeiss/Welch Allyn^®^). A simple algorithm was developed in which patients are initially triaged for a screening examination performed by either an ophthalmologist or an ophthalmic assistant, depending on whether the patient is above or below 50 years of age, respectively (Figure 1). This age cutoff was chosen based upon the strong association of glaucoma prevalence with increasing age and has been selected as a reasonable cutoff in other screening programs [10,18,19]. An ophthalmic assistant performed a flashlight examination of the anterior segment and distant direct ophthalmoscopy to detect media opacities on all subjects below 50 years of age. Patients with decreased visual acuity, corneal haze, cataract, shallow anterior chamber, or signs of PACG (e.g. iris atrophy, fixed pupils) are referred to the screening clinic ophthalmologist. FDT perimetry, applanation tonometry, and dilated funduscopic examination were performed on all patients 50 years of age or older as well as suspected glaucoma patients below 50 years of age. Patients with shallow anterior chambers as detected by penlight or slit lamp examination (Van Herick method) were preferentially referred to TEC for gonioscopy. Patients who were newly diagnosed with glaucoma at the screening clinic (or any other eye disease requiring further management) were also referred to TEC, where medication, and surgery if indicated, were provided free of charge through the generosity of private donors.

### 2. Glaucoma Awareness Week

Since 2004, TEC has hosted an annual Glaucoma Awareness Week in either March or April when weather conditions are most favorable for those patients traveling long distances. The event is co-sponsored by the Glaucoma Support Group of Nepal (GSGN) which was founded in 2003 and is a member of the World Glaucoma Patient Association (WGPA). Its members include glaucoma patients and their families, health care providers, and social workers. The objectives of the group are to educate glaucoma patients and their families about the disease, to promote public awareness of glaucoma, and to provide financial support for patients who are unable to afford treatment.

During this week glaucoma screening and any necessary treatment are provided free of charge. Compared to the one-day screening clinics, a more thorough standard eye examination is performed, including measurement of visual acuity using a Snellen chart, slit lamp examination, applanation tonometry, and fundus examination with a 90 diopter lens. All glaucoma suspects and newly diagnosed patients subsequently undergo disc photography, optical coherence tomography (OCT), and FDT perimetry. All patients who are newly diagnosed with glaucoma receive counseling from ophthalmic assistants and patient members of the GSGN, are enrolled in the GSGN and Patient Education Program lecture series, and are provided with free samples of medication (typically a topical beta-blocker, made possible in part by donations directly from pharmaceutical companies). Returning patients are also eligible to receive free interval evaluation, medication, and repeated counseling which emphasizes the importance of compliance with therapy. Patients who have more advanced disease requiring multiple medications or who live far away such that annual follow-up may be prohibitive are scheduled for free surgery (trabeculectomy). Ophthalmic assistants and students have created educational posters about glaucoma, and copies of a patient glaucoma handbook written in Nepali are available for purchase at a reasonable cost with proceeds reinvested in further educational and treatment efforts. In addition, a prize raffle has been held during previous Glaucoma Awareness Weeks to supplement fundraising efforts, with raffle tickets printed with educational messages such as risk factors for glaucoma. The GSGN publishes an annual report summarizing these activities and efforts, and the event has received local television, radio, and newspaper coverage, with patient attendance increasing in each of the four years since its inception. Approximately 90% of patients are from Kathmandu and the remaining 10% from villages in or near the Kathmandu valley.

### 3. Patient Education Program

Another important educational effort is the Patient Education Program, comprised of six lectures which take place at TEC on a selected Saturday morning every two months. The series is provided free of charge and is attended by members of the GSGN. Topics presented by ophthalmologists include an introduction to the GSGN, a discussion of glaucoma including risk factors, signs, symptoms, treatment modalities, diagnostic tools used in the diagnosis of glaucoma, instruction on how to properly administer eye drops, possible side effects of glaucoma medications, childhood glaucoma, new advances in glaucoma research, and the sharing of experiences by patients with glaucoma. At the end of each session attendees who have the financial means are encouraged to make a small donation towards a fund that supports treatment for poor patients throughout the year. In addition, some of the private donors mentioned in the "Screening Clinics" section are themselves glaucoma patients who attend our educational classes. These sponsors are again approached when new glaucoma patients are identified from screening clinics and are asked if they will further support medication for a year or more to one or more patients. Upon completion of the entire Patient Education Program a short quiz is administered, and those individuals who perform satisfactorily are able to become counselors for other glaucoma patients during Glaucoma Awareness Week. The series repeats each year, and starting in 2006 has been broadcast on a local FM radio channel to reach a wider audience.

## Results

From 2004 to 2007 screening at the annual Glaucoma Awareness Week has resulted in the diagnosis of 120 individuals with glaucoma, or 7.6% of total registrants (Table 1). Attendance has increased annually with a trend toward an increasing number of returning patients (from 145 in 2004 to 342 in 2007). There has also been a trend toward a decreasing percentage of newly diagnosed patients, though the absolute numbers have remained relatively stable (range 21 to 38). Data from the three one-day screening clinics in 2006 show that approximately 2–4% of patients 50 years of age or older per clinic were newly diagnosed with POAG (Table 2).

**Table 1:** Data from Glaucoma Awareness Week (2004–2007)

	**2004**	**2005**	**2006**	**2007**	**Total**
Total number registered	259	343	457	522	**1581**
Established glaucoma patients	145 (60%)	105 (30.6%)	231 (50.5%)	342 (65.5%)	**823 (52.1%)**
Newly diagnosed glaucoma patients	28 (10.8%)	38 (11.1%)	33 (7.2%)	21 (4.0%)	**120 (7.6%)**
Humphrey visual field analysis	129	137	235	203	**704**
FDT perimetry	123	53	42	147	**365**
Pachymetry	86	34	26	52	**198**
Optical coherence tomography	-	-	6	24	**30**
Disc photography	57	17	90	112	**276**
Bottles of medication distributed	51	87	264	368	**770**
Laser treatments	14	11	5	17	**47**
Patients supported for surgery	11	15	16	11	**53**

FDT = frequency doubling technology. Note that the quantity of medication distributed may not equal the number of glaucoma patients seen during a given year as not all established glaucoma patients received free medication in 2004 and 2005, and some patients received more than one sample in subsequent years. Similarly, some patients may have received both medication and another form of treatment (i.e. laser or surgery).

**Table 2:** Data from selected one day screening clinics (2006)

	**Bhotechaur**	**Lele**	**Badikhel**
Total number registered	318	180	298
Total number ≥ 50 years of age	99 (31%)	85 (47%)	99 (33%)
Patients diagnosed with POAG	2	1	3
Patients diagnosed with PAC	2	1	2
Glaucoma suspects referred to TEC	10	6	7
Number of glaucoma suspects who followed up at TEC	8	6	7
Glaucoma suspects ultimately diagnosed with POAG	2	1	1

**Total number diagnosed with POAG (incl. percent ≥ 50 years of age)**	**4 (4.0%)**	**2 (2.4%)**	**4 (4.0%)**

POAG = primary open-angle glaucoma; PAC = primary angle closure; TEC = Tilganga Eye Centre.

## Discussion and conclusion

Glaucoma is a public health issue of increasing importance as the global population increases in both age and number. While our limited data show that the programs described above are identifying undiagnosed glaucoma patients and benefiting individuals who would otherwise not be able to afford treatment, more data is needed to validate this approach. For example, an analysis of the effectiveness of educational activities could be performed through the use of surveys. It would also be important to know how many of the individuals referred to KEC for further management actually follow-up so that barriers to access can be better identified and addressed. A more current estimate of the prevalence of glaucoma in Nepal would also be helpful, as it is unclear if our Glaucoma Awareness Week data approximate regional glaucoma prevalence (such a study is currently being conducted in the city of Bhaktapur). Finally, and perhaps most importantly, more data is needed regarding medication compliance. Ensuring the continued availability of medication along with patient compliance are problems inherent in the management of glaucoma in the developing world, or any other chronic disease for that matter. Long term support for medical treatment remains a challenge because it involves soliciting continued support from donors. While annual free samples will not prevent blindness, they are an effective means to lessen the financial burden on patients and donors alike, as well as to motivate established patients to return for evaluation of disease progression and reinforcement of educational messages.

More extensive analysis of these programs would not only help to identify areas for improvement, but might also suggest different management approaches altogether. For example, interim results from the Collaborative Initial Glaucoma Treatment Study showed that patients randomized to either medical or surgical treatment for primary open-angle glaucoma had comparable outcomes at five years [20]. Such an approach has already been implemented in some parts of India[21]. While longer term data is needed before primary surgery can be more broadly recommended, the higher initial cost may ultimately prove to be justifiable in the long run for patients in developing countries such as Nepal who face greater obstacles to accessing health care resources. Similarly, prophylactic laser peripheral iridotomy (LPI) has been studied for the prevention and treatment of PACG in East Asian and Indian populations, though which patients would benefit most from this intervention is still being defined [22-24].

As mentioned previously, a simple and cost-effective screening plan for glaucoma has yet to be developed, a point emphasized by a 2006 Cochrane review that could not support population-based screening for primary open-angle glaucoma due to the fact that no randomized controlled trials could be identified.9 An analysis of data obtained from the Baltimore Eye Survey failed to demonstrate an acceptable balance of sensitivity and specificity in the study population for several tests (either separately or in combination) commonly used in the diagnosis of glaucoma, including tonometry, stereoscopic fundus photography, and Humphrey visual field analysis [11] More recent assessments of FDT perimetry as a screening tool in both developed and developing countries have been encouraging with good specificity for glaucoma; however, the test appears to have low sensitivity, and thus may be most beneficial as an adjunctive measure[25,26]. In light of the continuing challenges regarding accurate and cost-effective diagnosis of glaucoma in the general population it has been suggested that health care providers focus their efforts solely on case detection in high-risk individuals (e.g. older age, positive family history, or certain ethnic groups) instead [10,12,27]. This is an issue that will continue to evolve as our understanding of glaucoma, as well as diagnostic and therapeutic modalities, are further refined.

Like other developing countries, human resource and infrastructure development are critical to the success of future glaucoma screening and treatment efforts in Nepal; indeed, the development of such programs is meaningless if a society has neither the manpower nor the equipment and facilities with which to implement them. Presently, participation in the outreach programs described here is limited to villages near Kathmandu that have road access to and from TEC, facilitating initial evaluation and follow-up. Nepal has approximately 46 primary eye centers in some of the more remote areas of the country that are staffed by ophthalmic assistants trained to diagnose and treat minor eye diseases and refer patients to one of the 18 larger district eye hospitals when necessary. It is crucial that these providers are adequately trained in the diagnosis and effects of glaucoma as they may represent the only opportunity for case detection and education in their respective regions. TEC has previously conducted a glaucoma training workshop for ophthalmic assistants and continues to look for ways to improve such programs, as well as to expand training to include general ophthalmologists from the district eye hospitals where similar programs for screening, treatment, and education may be implemented if proven effective. Due in part to some of the challenges discussed above, glaucoma was not included as an avoidable cause of blindness in the VISION 2020 initiative, launched jointly in 1999 by WHO and the International Agency for the Prevention of Blindness (IAPB) [28]. Thus, it is incumbent upon eye care providers and public health officials to ensure that glaucoma remains a priority along with other more easily identifiable and treatable eye diseases. It is our hope that this task will be accomplished in part through the continued development, refinement, and validation of sustainable clinical and educational programs such as those described here.

## Competing interests

The authors declare that they have no competing interests.

## Authors' contributions

SST conceived and implemented the programs described herein. SST and IP supervised the data collection in the field. LC compiled the data into table format and contributed to the section on Glaucoma Awareness Week. KHK wrote the first draft of the manuscript, and SST, KHK and GVR revised the manuscript. All authors read and approved the final manuscript.

## Pre-publication history

The pre-publication history for this paper can be accessed here:


